# Supplementary material for: Genomic Characterization and Expressional Profiles of Autophagy-Related Genes (ATGs) in Oilseed Crop Castor Bean (Ricinus communis L.)
Source: Int J Mol Sci. 2020 Jan 15;21(2):562. doi: 10.3390/ijms21020562 (PMC7013546; doi:10.3390/ijms21020562)
Supplement: Supplementary file 1 [file ijms-21-00562-s001.pdf]

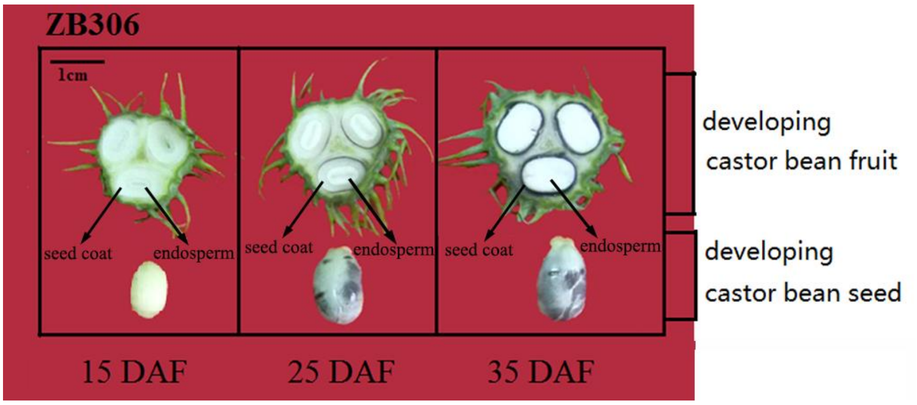

Supplemental Figure 1. Development process of castor seed(ZB306).DAF: day after fertilization.

Supplemental Table 1. The sequence of primers used for RT- qPCR

**Primer name**	**Primer sequences (5'→3')**
RcACTIN2 (F)	TTGGAATGGAAGCTGCAGGA
RcACTIN2 (R)	CCTTGCTCATACGGTCTGCAA
RcATG1a (F)	AAGTGGGCGGAGCAAGAA
RcATG1a (R)	AGTCCCAACAGCAAGAGCCT
RcATG1b (F)	TCTCAAATGCGAGGAACAATG
RcATG1b (R)	CAACCCCAGCCAGTCAAAG
RcATG1t (F)	ATGAACCTTGATTTTTGCGATG
RcATG1t (R)	GCTACTGCTTCACCCGTAATTCT
RcATG13a (F)	TTTCTCCAAACTTTTCTGACCC
RcATG13a (R)	TTCTGCTTTCTTGATTGACCG
RcATG13b (F)	GGAAACTTCTTGTGGGAGGC
RcATG13b (R)	AGGGGATACTGGGGTTGATG
RcATG13c (F)	TGTTGGTGCTCTTGTGCGTAT
RcATG13c (R)	TCTGTTCTTGGAGGCTAATGTTG
RcATG11 (F)	TCCAGTGACCGAGAAGTGTTTAT
RcATG11 (R)	AAGGCGTCATCTAAGGGGTG

RcATG101 (F)	TAACTCAGCAACCAAAAGCACA
RcATG101(R)	TGGAACAAAACCTCACGAAGAC
RcVPS34 (F)	AGCATCGCAGCATTACAAGC
RcVPS34 (R)	TGACGGAATAGCCAGCACAG
RcATG6 (F)	CTGCAATTTGAAATGGGCTCT
RcATG6 (R)	TTTGGAATCAACACCACGCT
RcVPS15 (F)	TGATATGAGGGTTGCGATGTG
RcVPS15 (R)	GCAGACGAGCAGAGTCCAGTAA
RcATG2 (F)	GTCCTCACCGTGTTGATCTAGC
RcATG2 (R)	ATACGTTGCCCCAGCCAT
RcATG9 (F)	AGTCTGGGTTTCACCATCTGTTT
RcATG9 (R)	CATCCATCCCACATTTAGCATT
RcATG18a (F)	TCTTCGCCGCCCATTTC
RcATG18a (R)	AACGGGTCGCAGTTGTAGATT
RcATG18b (F)	TCTTCCTGCAAGTACAACCAAAG
RcATG18b (R)	CACGATGAGCATCTATCTCACAAT
RcATG18c (F)	CTTTAGTCCCACCCCAGTCG
RcATG18c (R)	GCAGCAAAGCATCCATAATCC
RcATG18d (F)	GGTTTCCGGGTCTTTACTACG
RcATG18d (R)	GTTCGGTCCCACTGCCTACT
RcATG18e (F)	GTTTCATCTGGGGCATCTACTG
RcATG18e (R)	CATTGCTGGCATCGTCATCT
RcATG18f (F)	CCTGTAGGCTTTGAGCTGTCTT
RcATG18f (R)	ATCCTCTTCGGGCTTTTCC
RcATG18g (F)	CTGACAAGTGATGCTTCAAACG
RcATG18g (R)	GCCGATACTTTACAGTAACCCTCT
RcATG3 (F)	GCCAAAAGAATCTACGGGTGA

RcATG3 (R)	TCCAAAGTATGAGGGTATTGAGTG
RcATG4 (F)	GTGCCTTTGGTTCTTGGACTT
RcATG4 (R)	CCAGGTTTCCCACCCATAAT
RcATG5 (F)	GTTACTTGCCTCTGCTTATTCCC
RcATG5 (R)	TCAAACCAAACGGTGTCGG
RcATG7 (F)	TTGTAATGATTGGCGTAACTCG
RcATG7 (R)	CACCTTTTGGTCATCACTCTGG
RcATG8a (F)	TGCTGCATTGTTGTCTGCAAT
RcATG8a (R)	TTACTCTTGGAGTCCAAAGGTATTC
RcATG8b (F)	CATATTGCCTCCCACTGCTG
RcATG8b (R)	TCAGCTTGACTGCAACTCGAC
RcATG8c (F)	CTGCTATGATGTCTGCAATTTATGA
RcATG8c (R)	TTAGAATGATTCTCCAAATGTGTTCT
RcATG8d (F)	GTGCAATTATGTCTGCTATTTATGAAG
RcATG8d (R)	CTAGAGTGGAATCTCAGATCCAAAT
RcATG8e (F)	GTTCGAACATGATTTTGAGAAGAG
RcATG8e (R)	CAGGAATATCACTTCTTTCAGCCT
RcATG8f (F)	TACCTTGCCACAAACAGCTACTC
RcATG8f (R)	TTACAGATAGAAATTGGTAGCATGACC
RcATG8g (F)	AGCATTCGAGCAAAGACTCCA
RcATG8g (R)	GGCAGGTCCGTCTTTGAATATC
RcATG10 (F)	TAACTCAGGAGGAGCACCCAT
RcATG10 (R)	GCATCGCCAAGGAAAAGC
RcATG12 (F)	GGATATGCTGGCACTGTGTTATTC
RcATG12 (R)	ATCATCTTTTCCATCGAATCCC
RcATG16L (F)	AAAAGGTTACTGCACCAACACG
RcATG16L (R)	CCACAGACGAAGATTCCCATC
